# Identification and validation of a novel microRNA-like molecule derived from a cytoplasmic RNA virus antigenome by bioinformatics and experimental approaches

**DOI:** 10.1186/1743-422X-11-121

**Published:** 2014-07-01

**Authors:** Jiandong Shi, Zhiqing Duan, Jing Sun, Meini Wu, Bin Wang, Jing Zhang, Haixuan Wang, Ningzhu Hu, Yunzhang Hu

**Affiliations:** 1 Yunnan Key Laboratory of Vaccine Research & Development on Severe Infectious Diseases, Institute of Medical Biology, Chinese Academy of Medical Sciences & Peking Union Medical College, Kunming 650118, China; 2 Department of Life Science and Biotechnology, Kunming University, Kunming 650214, China

**Keywords:** Hepatitis A virus, Antigenome, MicroRNA-like molecule, Picornavirus

## Abstract

**Background:**

It is generally believed that RNA virus replicating in the cell cytoplasm would not encode microRNAs (miRNAs) due to nucleus inaccessibility. Recent studies have described cytoplasmic RNA virus genome-derived miRNAs in West Nile virus (WNV) and Dengue virus (DENV). However, naturally occurring miRNAs derived from the antigenome of a cytoplasmic RNA virus have not been described.

**Methods:**

Hepatitis A virus (HAV) was served as a model virus to investigate whether the antigenome of a cytoplasmic RNA virus would be processed into miRNAs or miRNA-like small RNAs upon infection. HAV antigenome was queried for putative miRNA precursors (pre-miRNA) with the VMir analyzer program. Mature miRNA prediction was performed using MatureBayes and Bayes-SVM-MiRNA web server v1.0. Finally, multiple experimental approaches, including cloning and sequencing-, RNAi-, plasmid-based miRNA expression- and luciferase reporter assays, were performed to identify and validate naturally occurring viral antigenome-derived miRNAs.

**Results:**

Using human HAV genotype IA (isolate H2) (HAVH2), a virally encoded miRNA-like small RNA was detected on the antigenome and named hav-miR-N1-3p. Transcription of viral pre-miRNA in KMB17 and HEK293T cells led to mature hav-miR-N1-3p production. In addition, silencing of the miRNA-processing enzyme Dicer or Drosha caused a dramatic reduction in miRNA levels. Furthermore, artificial target of hav-miR-N1-3p was silenced by synthesized viral miRNA mimics and the HAVH2 naturally-derived hav-miR-N1-3p.

**Conclusion:**

These results suggested that the antigenome of a cytoplasmic RNA virus could be processed into functional miRNAs. Our findings provide new evidence supporting the hypothesis that cytoplasmic RNA viruses naturally encode miRNAs through cellular miRNA processing machinery.

## Background

MicroRNAs (miRNAs) are small (approximately 22 nucleotide) regulatory non-coding RNAs that post-transcriptionally regulate gene expression by inhibiting the translation of mRNA transcripts or cleaving them [1-4]. MicroRNAs are encoded by cellular or viral genomes [5,6] and play a vital role in numerous cellular processes, including cell metabolism, viral infection, and antiviral immune response [7-9].

Indeed, a large number of miRNAs have been discovered in numerous organisms [1]. As an effective and economic regulatory strategy of gene expression, miRNAs are employed by viruses to regulate the expression of their own genes, host genes, or both [10,11]. Most viral miRNAs have been identified by traditional cloning strategy from virus-infected cells [12-15], yet others have been identified following computational prediction and hybridization analysis [15-17]. The current release (v20.0) of the miRNA registry [18,19] miRBase database lists 24,521 miRNAs, of which 493 viral miRNAs, indicating that diverse virus families encode miRNAs, including DNA and RNA viruses. It is noteworthy that the majority of known viral miRNAs are encoded by DNA viruses and only a few derived from RNA viruses. Indeed, DNA virus-encoded miRNAs are generally accepted, while miRNAs from RNA viruses, especially those that replicate in the cytoplasm, are controversial [10,11]. The rationale is that viruses with DNA genomes that replicate in the nucleus have access to cellular miRNA processing machinery; on the contrary, RNA viruses replicate in the cytoplasm and therefore would not encode miRNAs due to inaccessible miRNA processing machinery [10,11]. Retroviruses are generally believed to have the ability to encode miRNAs since a DNA stage is included in their infectious cycle [20]. It has been speculated that RNA viruses do not generate miRNAs, in order to avoid the adverse effects caused by the miRNA processing machinery [8]. Therefore, naturally occurring miRNAs derived from RNA viruses have not been widely acknowledged.

To date, RNA virus-encoded miRNAs have been identified only in few retroviruses, including the human immunodeficiency virus (HIV) [21,22], bovine leukemia virus (BLV) [20,23] and two cytoplasmic RNA viruses, namely the West Nile virus (WNV) and Dengue virus (DENV) [24,25]. Despite the theoretical barriers preventing cytoplasmic RNA viruses from encoding miRNAs, recent studies have confirmed that laboratory engineered RNA viruses, including the influenza virus, sindbis virus, and vesicular stomatitis virus (VSV), are capable of expressing miRNA-like small RNAs [26-30]. Furthermore, identification and validation of WNV and DENV derived miRNAs demonstrated that cytoplasmic RNA viruses indeed encode miRNAs through cellular miRNA processing machinery. Therefore, we hypothesized that the antigenome of a cytoplasmic RNA virus could be processed into miRNA-like small RNAs by the cellular miRNA processing machinery, similar to virus genomes. To test this hypothesis, we used a strategy that combined computational prediction and experimental validation with hepatitis A virus (HAV), a typical cytoplasmic RNA virus, searching for putative miRNA-like small RNAs. Although high-throughput sequencing has been widely used to characterize miRNA profiles and discover novel miRNAs in a variety of organisms, experimental screening of viral miRNAs by high-throughput sequencing of a large number of cDNA clones from infected cells is technically challenging, time consuming and likely incomplete. More importantly, viral gene expression displays highly constrained tissue-, time-, and replication state-specific patterns [17]. The above mentioned drawbacks could be efficiently overcome by a strategy combining computational prediction and experimental identification.

In the present study, HAV strain H2 was chosen as a model virus to investigate whether the antigenome of a cytoplasmic RNA virus can be processed into miRNA-like small RNAs, for the following reasons: first, the H2 strain of HAV is highly attenuated and does not cause disease in humans, which makes it an ideal model for studying HAV life cycle and virus-host interactions. In addition, HAV is a typical cytoplasmic RNA virus with approximately 7.5 kb genome and its antigenome is generated during viral replication. Finally, HAV has several unique biological characteristics that distinguish this virus from other members of the picornavirus family, including slow replication and persistent infection in most HAV/cell culture systems without a cytopathic effects [31-33]. Based on these HAV characteristics, we examined if its antigenome would encode miRNA-like small RNAs to regulate viral replication and virus-host interactions. Through computational prediction and experimental approaches, we demonstrated the generation and expression of a novel HAV antigenome-encoded miRNA in infected cells. This study provides new evidence supporting the hypothesis that a cytoplasmic RNA virus can encode functional miRNAs through cellular miRNA processing machinery. In addition, our findings provide a basis for further works assessing the roles of the HAV antigenome-encoded miRNA during virus infection and virus-host interactions. To our knowledge, this is the first study to identify and validate miRNAs on the antigenome of a cytoplasmic RNA virus.

## Results

### Putative pre-miRNA stem-loop structures and mature miRNAs in hepatitis A virus antigenome

**Figure 1:**
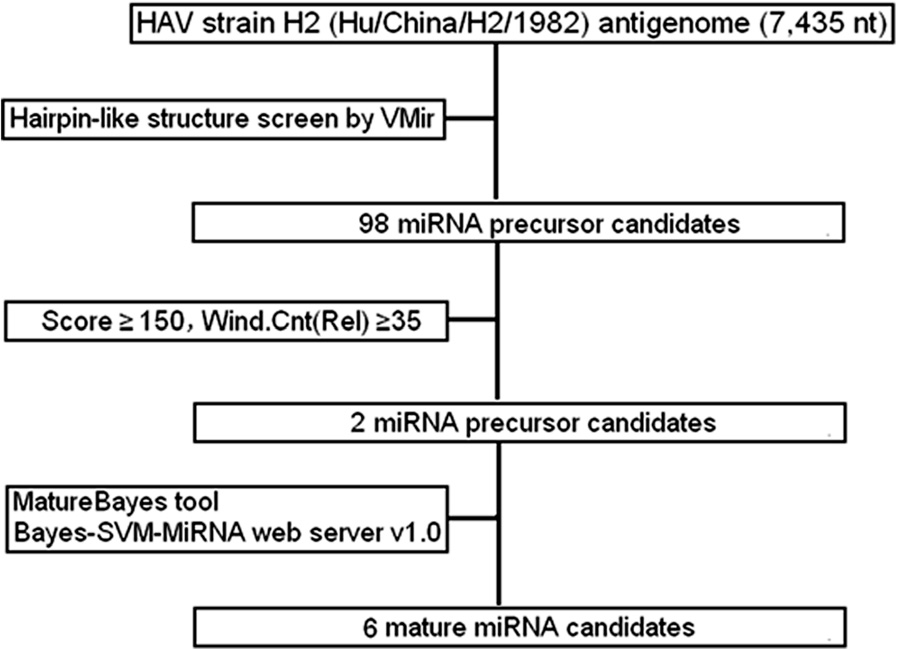
**Flowchart of HAV antigenome-encoded miRNAs prediction procedure.** The VMir analyzer program was used to predict putative HAV antigenome-encoded pre-miRNA stem-loop structures, and the MatureBayes tool and Bayes-SVM-MiRNA web server v1.0 were used to predict mature miRNA sequences.

**Figure 2:**
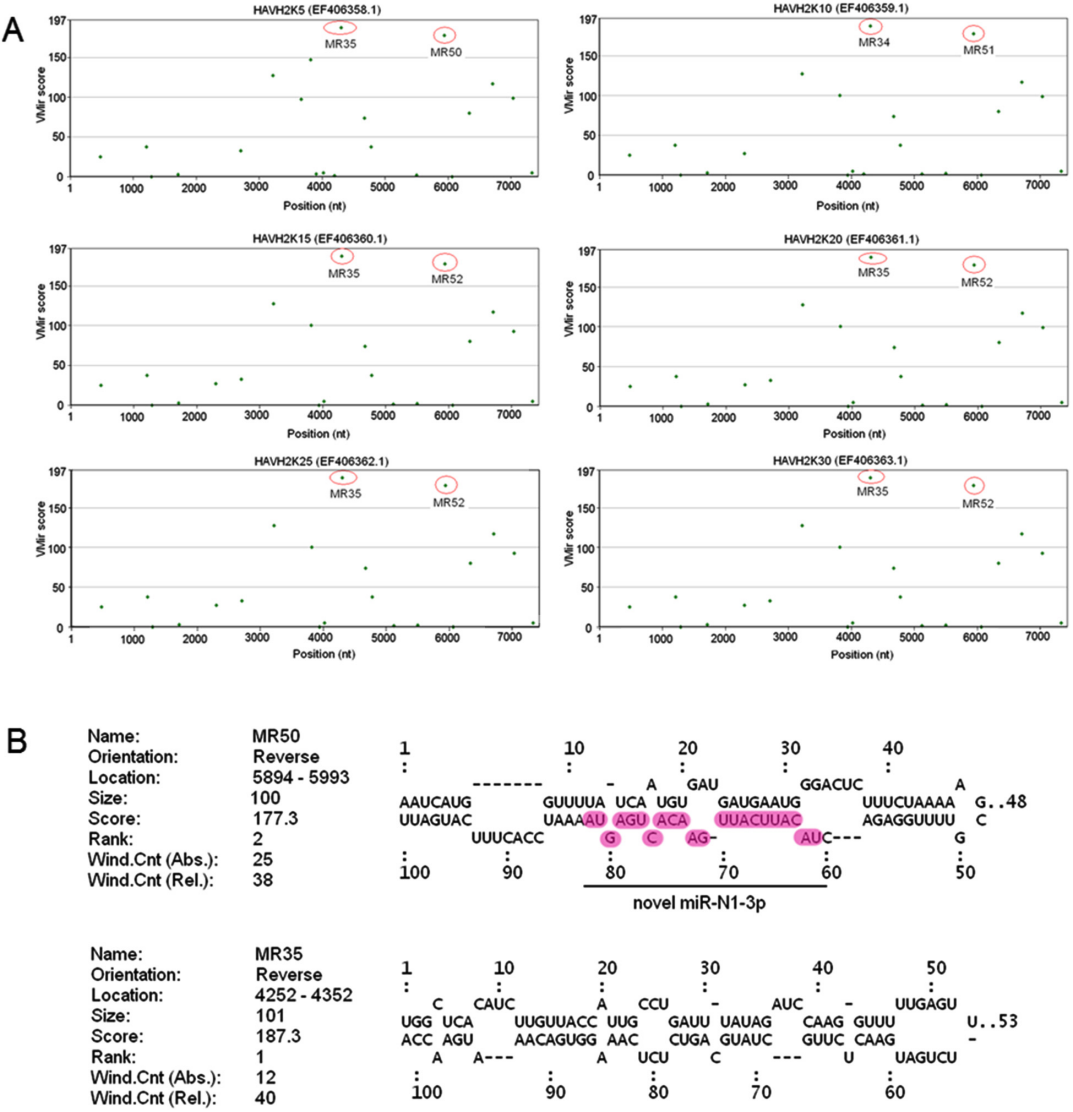
**VMir prediction of HAV antigenome-encoded pre-miRNAs. (A)** The candidate pre-miRNA with high scores is circled in red. The VMir analyzer program was used to predict candidate pre-miRNAs in the HAV antigenome with a cutoff score of 150 and a cutoff window counts of 35. The *y*-axis represents VMir scores, while the *x*-axis indicates the nucleotide position. **(B)** Secondary structures of the predicted candidate HAV pre-miRNAs. Mature miRNA that was experimentally validated in this study is highlighted and indicated as “novel miR-N1-3p”.

Computational prediction represents an effective strategy to identify novel miRNAs that can be further examined and validated by experimental approaches. Therefore, it is widely utilized to discover novel low abundance and temporal or tissue-specific miRNAs from various organisms [34]. In the HAV replication cycle, the antigenome is synthesized with a genome template [33]. RNA virus antigenomes distributed in the cytoplasm form various secondary structures and constitute potential pri/pre-miRNAs to generate miRNAs. In order to query whether HAV antigenome could be folded into putative pre-miRNA stem-loop structures, we analyzed its putative miRNA-encoding capacity. Using the VMir analyzer program [16,35,36], 98 sequences located in HAV antigenome with potential stem-loop structures were found (Figure 1), suggesting the potential miRNA-encoding capacity of the HAV antigenome. Moreover, to identify conserved pre-miRNAs among different cell-adapted passages of HAV strain H2, all antigenomes from six H2 strain passages were screened using the VMir analyzer program. Two pre-miRNA candidates, MR50 and MR35, which were conserved among different HAVH2 cell-adapted passages (GenBank accession no. HAVH2K5: EF406358.1, HAVH2K10: EF406359.1, HAVH2K15: EF406360.1, HAVH2K20: EF406361.1, HAVH2K25: EF406362.1, and HAVH2K30: EF406363.1) with VMir score ≥150 and window counts ≥35, were selected (Figure 2A). More importantly, other pre-miRNA candidates with identical sequences and stem-loop structures as MR50 or MR35 were obtained in additional five antigenomes from HAV passages (Figure 2B), suggesting that MR50 and MR35 pre-miRNA sequences and structures were completely conserved among different HAV cell-adapted passages. Thus, MR50 and MR35 were considered putative pre-miRNA candidates, and their imperfect stem-loop secondary structures have typical characteristics of pre-miRNAs. VMir analyzer program prediction revealed that MR50 was derived from the cell-adapted HAVH2K5 passage (GenBank accession no. EF406358.1) and located on the antigenome (from 5894 to 5993 nt, 100 nt), with a score of 177.3 and -23.6 minimal free energy. The other pre-miRNA, MR35, was located on the antigenome (from 4252 to 4352 nt, 101 nt) with a score of 187.3 and -23.0 minimal free energy. Simultaneously, their secondary structures were predicted by RNAFold algorithms with typical imperfect stem-loop structures of pre-miRNAs (Figure 2B). Furthermore, considering about 22 nucleotides (nt) in length of the mature miRNAs spliced from pre-miRNAs, we predicted the potential mature miRNA candidates by the MatureBayes tool [37] (http://mirna.imbb.forth.gr/MatureBayes.html) and Bayes-SVM-MiRNA web server v1.0 (http://wotan.wistar.upenn.edu/BayesSVMmiRNAfind/) (Additional file 1). MatureBayes tool prediction revealed 4 putative mature miRNA candidates with 22 nt, namely MR50-1, -2 and MR35-1, -2, and derived from 5p or 3p of two putative pre-miRNAs MR50 and MR35 (Table 1). Prediction results from Bayes-SVM-MiRNA web server v1.0 yielded 2 putative mature miRNA candidates with 21 nt. They were derived from 3p and 5p of the MR50 or MR35 stem-loop structure (Table 1) and termed as MR50-3, MR35-3. Overall, using computational approaches, we found that the antigenome of HAVH2 putatively encodes 2 pre-miRNAs and 6 mature miRNA candidates. These results provide evidence from bioinformatics perspective that HAV antigenome might encode miRNAs, and lay foundation for further experimental validation of the candidate miRNAs.

**Table 1:** Information of the predicted HAV antigenome-encoded miRNAs

**HAV miRNA**^**a**^	**miRNA sequence (5'-3')**	**Length (nt)**	**3p /5p**	**Position**^**b **^**(nt)**	**Prediction tool**^**c**^
MR50-1	UACAUUCAUUGAACACUGAGUA	22	3p	5912 to 5933	MatureBayes
MR50-2	UAAAAAGCGUUUUGGAGACUAC	22	5p	5931 to 5952	MatureBayes
MR50-3	GAUGAUGAAUGGGACUCUUUC	21	5p	5953 to 5973	Bayes-SVM-MiRNA web server.
MR35-1	GAACUCUUGCUAUGCAGUCUCU	22	3p	4272 to 4293	MatureBayes
MR35-2	UGAUUUAUAGAUCCAAGGUUUU	22	5p	4306 to 4327	MatureBayes
MR35-3	CUAUGCAGUCUCUCAAAGGUG	21	3p	4264 to 4284	Bayes-SVM-MiRNA web server.

^a^The miRNAs (miR) derived from a single miRNA stem-loop precursor are indicated by a 5p (5' arm) or a 3p (3' arm) suffix.

^b^Genomic positions are provided based on the published HAV genome sequence (GenBank accession no. EF406358.1).

^c^Prediction strategy of each mature miRNA sequence is shown in table.

### A HAV antigenome-derived small RNA is generated and expressed in infected cells

**Figure 3:**
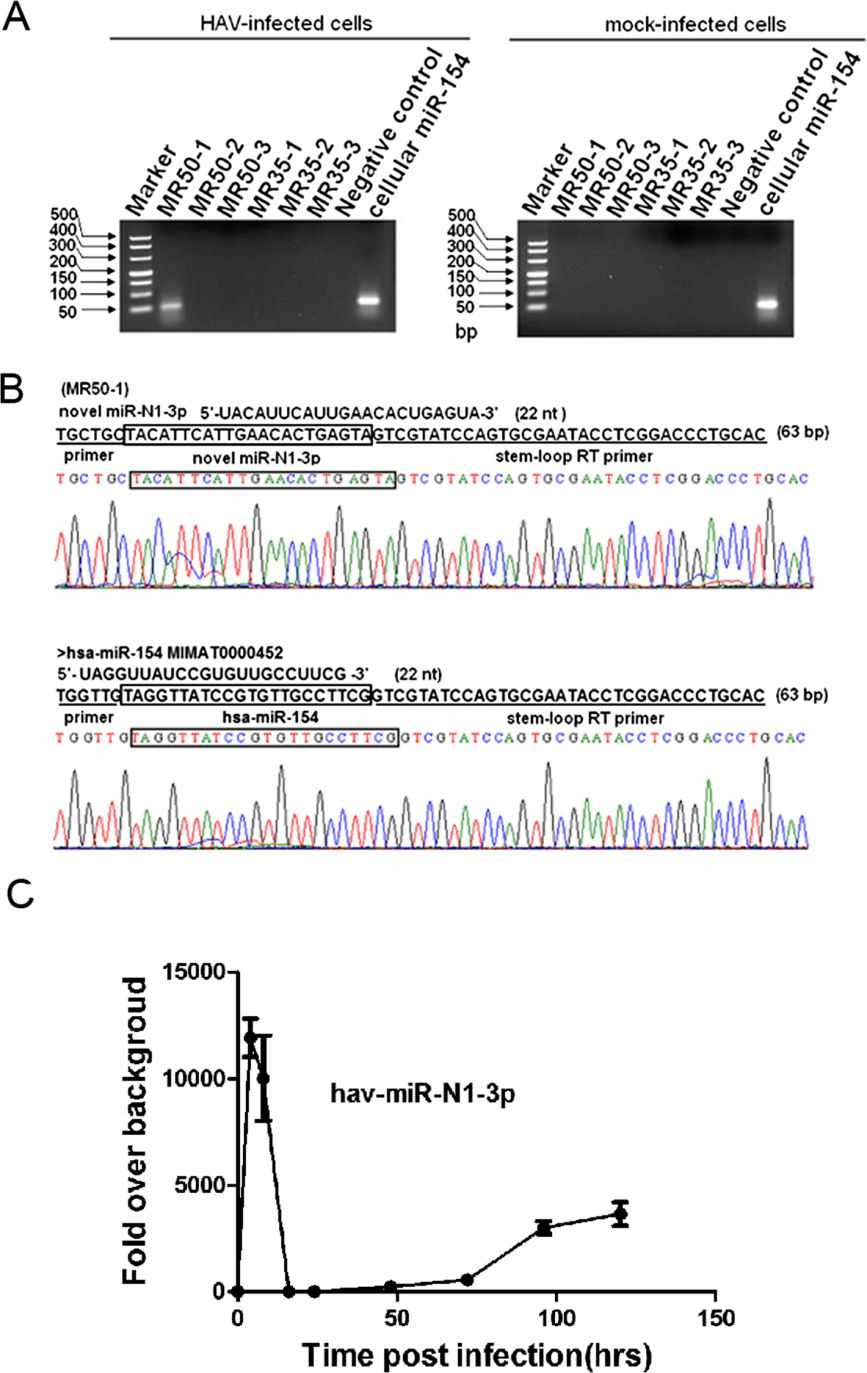
**Cloning and expression validation of the predicted miRNAs in HAV-infected and mock-infected cells. (A)** A HAV antigenome-encoded miRNA was detected in the HAV-infected cells. 4% agarose gel electrophoresis analysis showed stem-loop RT-PCR products with approximately 63 bp specific bands. Cellular miR-154 was used as a positive control when performing agarose gel electrophoresis. **(B)** Sequencing results for the novel HAV miRNA and primer sequences are underlined in the sequencing maps. **(C)** Time course analysis of hav-miR-N1-3p in KMB17 cells. KMB17 cells were infected with HAV at a MOI of 0.5 TCID_50_/cell. Total RNA was extracted at 0, 4, 8, 12, 24, 48, 72, 96 and 120 hpi. and measured by qRT-PCR to quantify the expression of hav-miR-N1-3p during infection. All values are presented as changes in expression relative to uninfected cells after normalization to values of snU6 RNA.

In order to ascertain whether the predicted miRNA candidates were indeed produced and expressed in HAV infected cells, stem-loop RT-PCR as well as PCR-based directed cloning and sequencing analyses were performed as previously described [38-40]. As shown in Figure 3A, a novel miRNA candidate, MR50-1, was successfully amplified specifically by stem-loop RT-PCR with a band of 63 bp in HAV-infected cells. In contrast, no corresponding band was observed in mock-infected cells. Considering the possibility that some predicted miRNAs might not be detected due to their low abundance, we performed a more sensitive qRT-PCR analysis. The results showed that MR50-1 was detected with a specific signal in HAV-infected cells; no signals for other miRNA candidates were detected (data not shown). More importantly, no specific signals for the predicted miRNA candidates except a cellular miR-154 (a well-annotated miRNA), were detected from RNA isolated from mock-infected cells. These results indicated that the miRNA candidate MR50-1 was indeed generated and expressed in HAV-infected cells. Furthermore, in order to determine the exact sequence of the amplified miRNA, a PCR-based directed cloning and sequencing analysis were performed. Interestingly, the amplified sequence of MR50-1 was completely consistent with the predicted MR50-1 (Figure 3B). Using the above methods, we obtained the exact sequence of MR50-1 and confirmed its expression during viral infection. MR50-1 represented a novel miRNA candidate and was named hav-miR-N1-3p. Although the 3p arm of the miRNA MR50 was detected, its 5p arm, including MR50-2 and MR50-3, could not be detected in our analysis. In addition, the sequencing results showed that the cellular miR-154 that served as a positive control, was also amplified with a band of 63 bp, suggesting that stem-loop RT-PCR is a highly specific and sensitive method for the detection of miRNA expression. To characterize specific temporal expression pattern of hav-miR-N1-3p throughout the infection, we performed a highly sensitive and specific qRT-PCR analysis. As shown in Figure 3C, the hav-miR-N1-3p reached to a maximum plateau level at 4 hours post-infection, then decreased gradually and maintained a stable and low level during infection, whereas the value measured for cells non-infected remained at or close to backgrounds levels. The results suggested that the generation of hav-miR-N1-3p occurred in the initial stage of infection. As the infection progresses, hav-miR-N1-3p maintained a stable range with a low level. Notably, after we detected hav-miR-N1-3p in infected cells, we performed a northern blot assay to further confirm the presence of pre- and mature hav-miR-N1-3p and characterize its specific temporal expression pattern throughout the infection. Regrettably, we did not detect signals of the pre- and mature miRNA in all indicated time points (data not shown). The major reason for this result is possible that low and inefficient viral replication lead to low miRNA copy number and low miRNA expression level. Therefore, low expression miRNA cannot be detected by northern blot owing to its limited detection sensitivity. Taken together, these results confirmed that the novel miRNA candidate hav-miR-N1-3p encoded by antigenome of HAV is indeed produced and expressed in HAV-infected cells.

### Depletion of Dicer decreases the expression of HAV antigenome-encoded hav-miR-N1-3p

**Figure 4:**
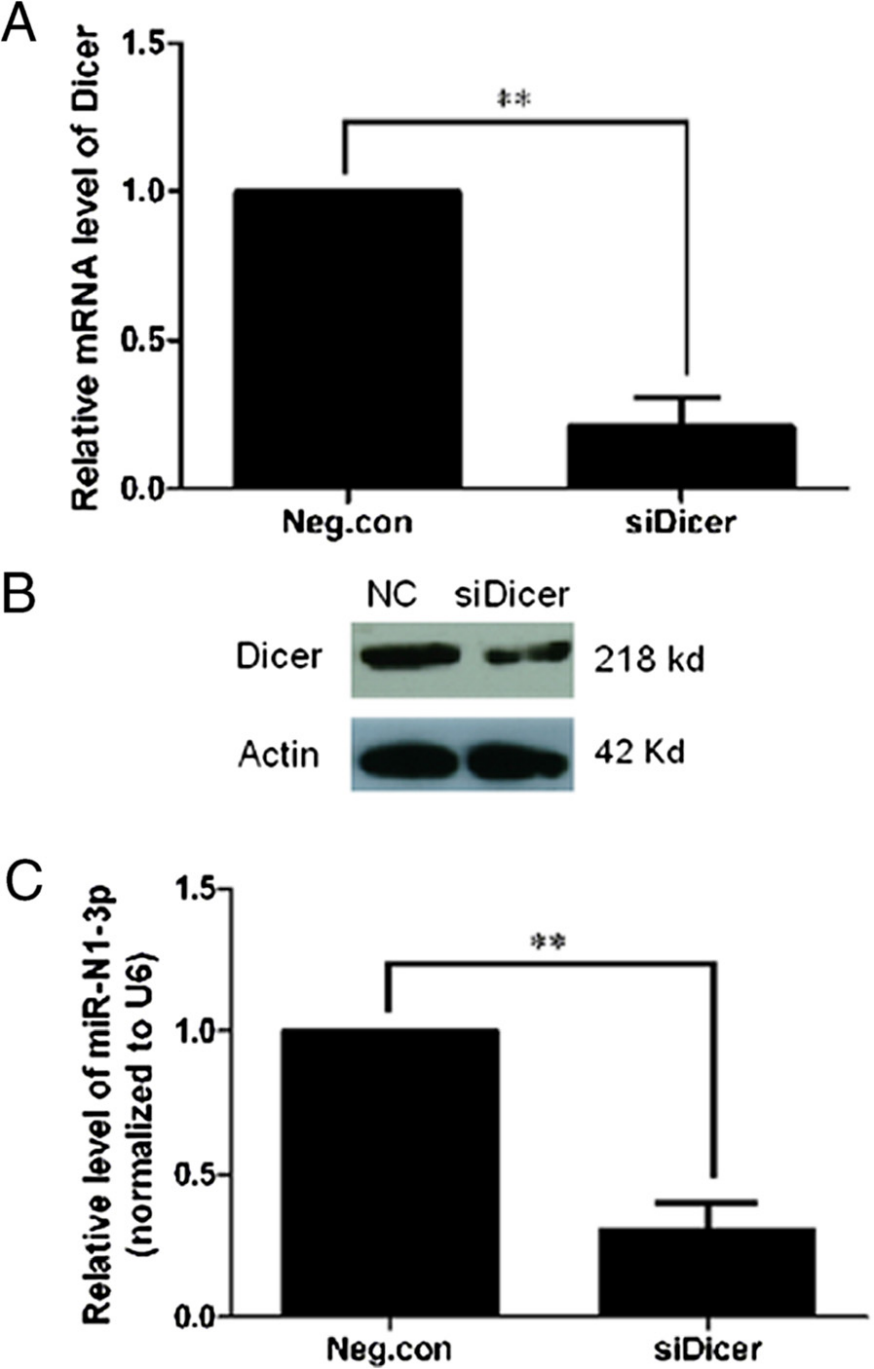
**Knockdown of Dicer reduces the expression of HAV antigenome-encoded hav-miR-N1-3p. (A)** Dicer mRNA was knocked down by RNAi and confirmed by qRT-PCR. **(B)** Dicer protein was knocked down by RNAi and confirmed by Western blot. Actin was used as a loading control. **(C)** Expression difference analysis of HAV antigenome-encoded hav-miR-N1-3p between Dicer-silencing and negative siRNA-transfected cells. HAV antigenome-encoded hav-miR-N1-3p expression changes were calculated relatively to U6 snRNA, and cellular miR-154 was used as a positive control. Significant difference in expression levels were detected by Student’s *t*-test (*P < 0.05 and **P < 0.01).

Dicer is well known as a key protein in the canonical cellular miRNA processing pathway, which is responsible for ultimately processing pre-miRNA into mature miRNA/miRNA* duplex [41]. It is well established that reduction of vital components of the miRNA processing pathway leads to an attenuation of miRNA steady-state levels [42]. Therefore, to ascertain whether hav-miR-N1-3p is a product of the cellular Dicer processing pathway, a depletion of Dicer was carried out using siRNA-based RNAi approach followed by infection with HAV. Silencing efficiency of Dicer mRNA and protein were monitored by qRT-PCR and Western blot. As shown in Figure 4A, Dicer mRNA expression levels were reduced dramatically after transfection with Dicer-specific siRNAs, compared with negative control siRNA-treated cells. Furthermore, Dicer protein levels were reduced significantly in Dicer-specific siRNA group, compared with negative control siRNA-treated cells (Figure 4B). In addition, the expression difference in viral miRNA between Dicer-knockdown cells and negative control cells was evaluated by qRT-PCR at 24 hours post-infection. As shown in Figure 4 C, a significant reduction of relative viral miRNA levels was observed in Dicer-knockdown cells, compared with negative control siRNA-transfected cells (*P* < 0.01). These results were in agreement with a report by Hussain *et al*. [24]. Additionally, we examined the effect of Dicer alteration on viral replication by qRT-PCR. Interestingly, alteration of Dicer expression did not affect the total amounts of viral RNAs (data not shown). These data demonstrated that depletion of Dicer decreased the expression of HAV antigenome-encoded miR-N1-3p. Collectively, these data suggested that hav-miR-N1-3p is a product of the cellular Dicer processing pathway, rather than random degradation products of viral antigenomes. Notably, we also demonstrated that silencing of Drosha reduced hav-miR-N1-3p expression (Additional file 2), which suggested that hav-miR-N1-3p might be a Drosha-processing product.

### Transcription of HAV pre-miRNA in KMB17 and HEK293T cells from plasmid results in mature hav-miR-N1-3p production

**Figure 5:**
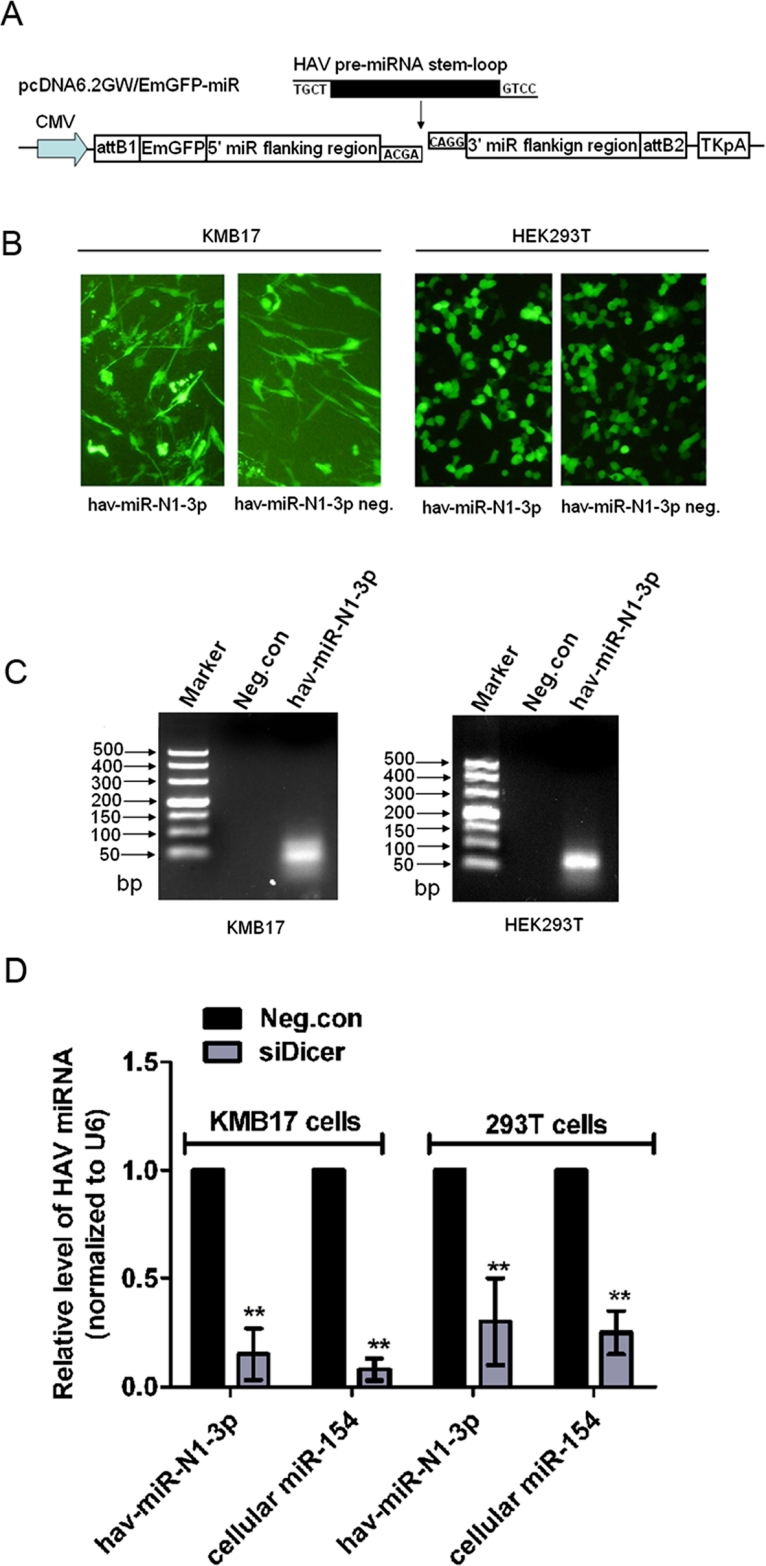
**Detection of hav-miR-N1-3p from plasmid-expressed precursor miRNA (pre-miRNA). (A)** Construction of miRNA expression plasmid. Each pre-miRNA sequence was cloned into a pcDNA6.2-GW/EmGFP-miR expression vector. The vector with scrambled sequences was used as a negative control. **(B)** KMB17 and HEK293T cells were transfected with miRNA expression plasmid, and strong green fluorescence was observed in both transfected cell lines (100 ×). **(C)** Detection of mature miRNA hav-miR-N1-3p expression. Small RNA was harvested at 24 hours post-transfection, and mature hav-miR-N1-3p was detected by stem-loop RT-PCR in both transfected cells. **(D)** Plasmid-expressed hav-miR-N1-3p was reduced in Dicer-deficient KMB17 and HEK293T cells compared with Dicer-non-deficient negative control cells. Significant difference in expression levels were detected by Student’s *t*-test (*P < 0.05 and **P < 0.01).

Because the viral hav-miR-N1-3p confirmed by cloning and sequencing may be a random degradation product from the viral antigenome, we carried out a plasmid-derived miRNA expression analysis to exclude this possibility. In order to determine whether viral hav-miR-N1-3p was derived from the MR50 precursor, the predicted HAV pre-miRNA MR50 was cloned into the mammalian expression plasmid pcDNA6.2-GW/EmGFP-miR to generate recombinant pcDNA6.2-GW/EmGFP-hav-pre-miRNA-50, which was transfected into KMB17 and HEK293T cells (Figure 5A). Total RNA isolated from cells transfected with the expression plasmid of viral hav-miR-N1-3p or the negative control plasmid with scrambled sequences (Invitrogen) was subjected to a stem-loop RT-PCR analysis. Green fluorescent protein (EmGFP) with strong intensity was observed by fluorescence microscopy in both transfected cell groups (Figure 5B). Moreover, RT-PCR analysis revealed that viral hav-miR-N1-3p was expressed with an apparent band of 63 bp in KMB17 and HEK 293 T cells transfected with the hav-miR-N1-3p expression plasmid pcDNA6.2-GW/EmGFP-hav-pre-miRNA-50 (Figure 5C). In contrast, no bands were detected in cells transfected with the negative control plasmid. More importantly, sequence analysis of the PCR product indicated that the PCR amplicon sequence was completely consistent with viral hav-miR-N1-3p (data not shown). Furthermore, in order to determine whether viral hav-miR-N1-3p expressed by pcDNA6.2-GW/EmGFP-hav-pre-miRNA-50 was generated from the predicted MR50 precursor by the Dicer processing pathway, we performed a silencing analysis of Dicer using RNAi approach in KMB17 and HEK293T cells. Knockdown of Dicer was monitored and confirmed by qRT-PCR and Western blot (data not shown). Dicer-deficient and Dicer-non-deficient KMB17 and HEK293T cells were transfected with pcDNA6.2-GW/EmGFP-hav-pre-miRNA-50, and qRT-PCR analysis was carried out to measure the relative levels of viral hav-miR-N1-3p. As shown in Figure 5D, a dramatic reduction in relative viral hav-miR-N1-3p levels was found in Dicer-deficient cells, compared with Dicer-non-deficient cells (*P* < 0.01). Taken together, these data indicated that viral hav-miR-N1-3p is processed from the corresponding MR50 precursor by the Dicer processing pathway. Notably, when Dicer was knocked down by specific siRNAs, viral hav-miR-N1-3p and cellular miR-154 were all reduced significantly, compared with Dicer-non-deficient KMB17 or HEK293T cells (Figure 5D). These findings suggested that viral hav-miR-N1-3p expressed by the plasmid pcDNA6.2-GW/EmGFP-hav-pre-miRNA-50 was produced in a similar way to cellular miR-154. Furthermore, these results indicated that viral hav-miR-N1-3p is indeed derived from the splicing of the predicted MR50 precursor by the cellular miRNA processing pathway rather than the random degradation products of viral antigenome RNAs.

### HAV antigenome-encoded hav-miR-N1-3p is biologically functional and mediates post-transcriptional gene silencing (PTGS)

**Figure 6:**
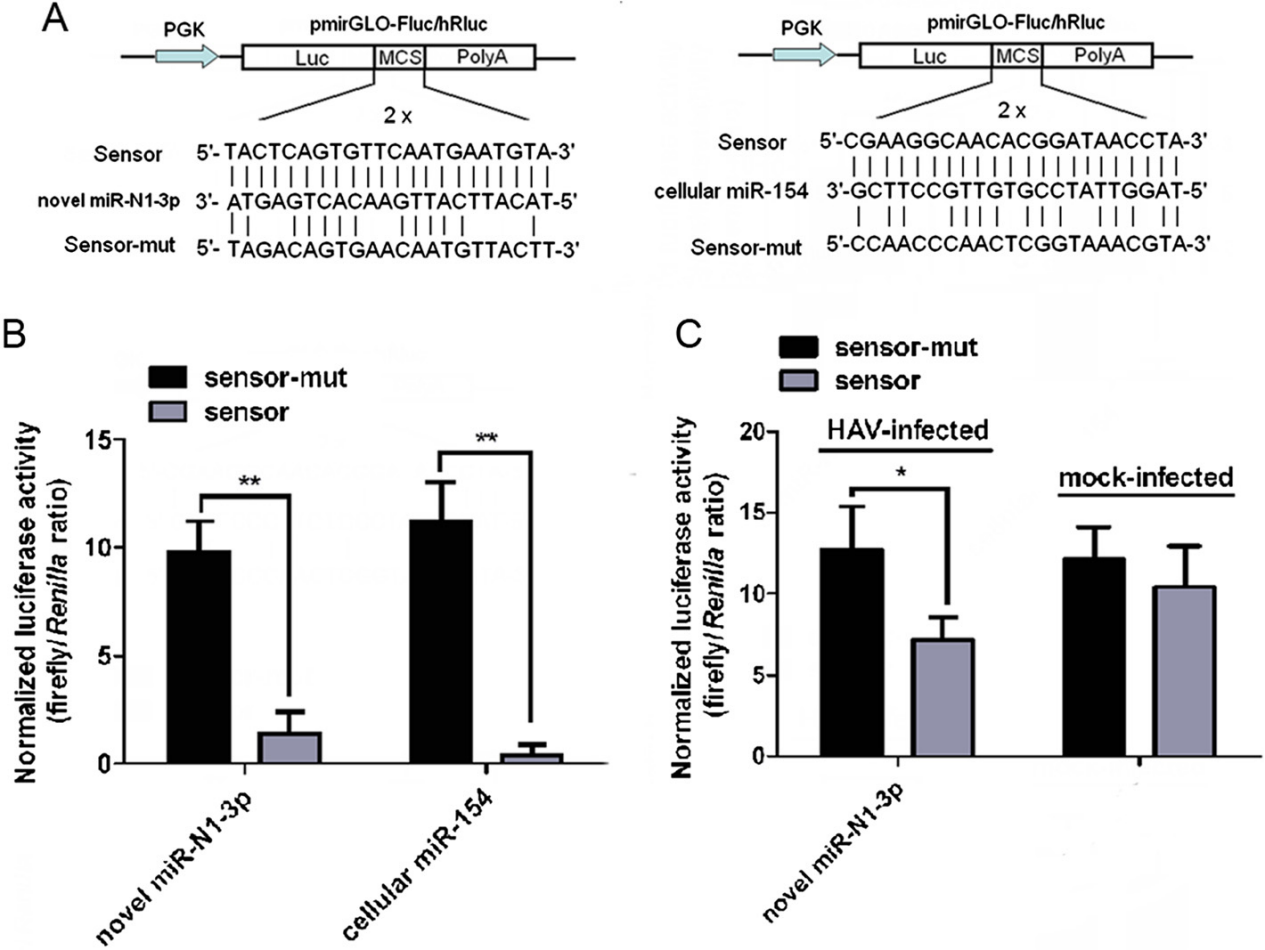
**HAV antigenome-encoded hav-miR-N1-3p is a biologically functional miRNA. (A)** Construction of sensor and sensor-mut plasmids. Sensor construct with two tandem repeats of artificial target sites perfectly complementary to the miRNA sequence were inserted into the 3' UTR of the luciferase gene in pmirGLO vector (sensor), and mutant construct with two tandem repeats of mutated nucleotides in the artificial target sites (sensor-mut). **(B)** Silencing activity of exogenous viral hav-miR-N1-3p in HEK293T cells transfected with viral miRNA mimics. **(C)** Silencing activity of endogenous viral hav-miR-N1-3p in HAV-infected and mock -infected cells. Luciferase activity was assessed 48 hours after transfection. A cellular miR-154 served as a positive control and the mutated plasmid was used as a negative control. For all treatments, relative luciferase activity was expressed as ratio of firefly to *Renilla* luciferase activity. Error bars indicate standard deviation obtained in three replicates, and significant differences are indicated with *at *P* < 0.05 and **at *P* < 0.01.

One of the known biological characteristics of genuine miRNAs is that they can post-transcriptionally silence gene expression [6,24] namely PTGS. Therefore, in order to determine whether viral hav-miR-N1-3p is functionally active, a dual-luciferase reporter assay using pmirGLO-hav-miR-N1-3p-sensor or pmirGLO-hav-miR-N1-3p-sensor-mut plasmid was carried out in HEK293T and KMB17 cells (Figure 6A). First, we tested the silencing activity of exogenous viral miR-N1-3p on artificial target of viral miR-N1-3p with synthesized viral miRNA mimics in HEK293T cells. As shown in Figure 6B, relative luciferase activity was significantly reduced when co-transfected with hav-miR-N1-3p miRNA mimics and pmirGLO-hav-miR-N1-3p-sensor plasmid, reaching up to 85.70 ± 10.21% (Figure 6B; *P* < 0.01) after silencing compared to the negative control (mutated sensor plasmid). In addition, a lower silencing effect for cellular miR-154 (96.40 ± 4.46%) was observed (Figure 6B; *P* < 0.01). These data indicated that synthesized viral hav-miR-N1-3p mimics is able to silence the expression of the luciferase gene with inserted artificial target of viral hav-miR-N1-3p and induced PTGS. Furthermore, in order to determine whether HAV naturally derived miR-N1-3p is able to silence the luciferase gene with inserted or mutated artificial target of viral hav-miR-N1-3p, we assessed its silencing on sensor and sensor-mut plasmid in HAV-infected and mock-infected KMB17 cells. As shown in Figure 6C, a dramatic decrease in relative luciferase was observed with the sensor plasmid, compared to the negative control (mutated sensor plasmid), in HAV-infected cells. Silencing efficiency of viral hav-miR-N1-3p on its artificial target reached 43.22 ± 11.04% (Figure 6C; *P* < 0.05). However, no obvious difference for sensor and sensor-mut negative controls was observed in mock-infected cells (Figure 6C). These data suggested that hav-miR-N1-3p is capable of silencing the expression of the luciferase gene with inserted artificial targets of viral hav-miR-N1-3p, inducing PTGS. Overall, these results suggested that HAV antigenome-encoded hav-miR-N1-3p is a functionally active miRNA molecule.

## Discussion

Although two novel miRNAs have been experimentally identified in WNV and DENV genomes, respectively [24,25], the controversy remains as whether a virus with RNA genome that replicates in the cytoplasm can naturally encode functional miRNAs or miRNA-like small RNAs. The present study identified a novel miRNA-like small RNA generated from the antigenome of HAV strain H2 using computational prediction and experimental approaches. These findings provide new evidence for a cytoplasmic RNA virus encoding functional miRNAs. In this study, bioinformatics analysis played a crucial role in the discovery of the novel viral miRNA. However, computational prediction does not necessarily implies that the predicted miRNAs are indeed generated and expressed in virus-infected cells. Thus, experimental validation of predicted miRNAs is required and needed. Several techniques are available for this validation including cloning, RT-PCR and deep sequencing. A PCR-based directed cloning and sequencing assay for the predicted miRNAs was performed in this study. The results indicated that the miRNA candidate MR50-1 was successfully amplified and its actual sequence was revealed by sequencing. Obviously, computational prediction followed by experimental validation constitutes an effective and fast strategy for discovery and identification of novel and rare, time-, and tissue-specific miRNAs. Several studies have previously employed this strategy to identify a few miRNAs in numerous viruses [43-45]. Using the VMir analyzer program, the simian virus 40 (SV40), Merkel cell polyomavirus (MCV) and murine polyomavirus (PyV) have been confirmed to encode one or more miRNAs [5,6], which suggested that VMir analyzer program is an effective tool for searching new viral miRNA-like small RNAs.

As a RNA virus, HAV presents numerous mutations over its genome and antigenome during cell culture adaption [46-49], suggesting that the viral genome is not exactly the same among different cell-adapted passages and various HAV strains. Thus, it is difficult to find miRNAs that are completely conserved among different viral strains due to genome mutations. However, it is possible that some miRNAs are completely conserved among diverse viral cell-adapted passages. In this study, a specific HAV strain H2 was selected as a model virus. Using the VMir software, two miRNA precursors, MR50 and MR35, were found and shown to be completely conserved among different viral cell-adapted passages. These findings indicated that MR50 and MR35 stem-loops are always formed by the HAV strain H2 during infection. However, MR50 and MR35 are not conserved in other HAV strains. When other HAV strains were examined, other distinct pre-miRNA stem-loops were obtained, not MR50 and MR35. Theoretically, the HAV genome might form extensive RNA structures like antigenome, thus can be processed into miRNA-like small RNAs by the cellular miRNA machinery. Thus, we analyzed the genome of HAV with the VMir software, and obtained three pre-miRNAs, including MD5, MD61 and MD81, located in the viral genome coding region. These observations suggested that the RNA secondary structures of HAV strain H2 genome might be processed into miRNA-like small RNAs. It is noteworthy that the cellular miRNA machinery cannot distinguish viral genome from antigenome. However, the miRNA machinery can segregate stem-loop structures from non-stem-loop structures. This suggested that candidate pre-miRNA stem-loop structures, whether from viral genome or antigenome, might be processed into miRNA-like small RNAs. In the present study, we aimed to determine whether a cytoplasmic RNA virus antigenome can be processed into miRNA-like small RNAs. Thus, HAV genome-derived miRNAs are beyond the scope of the present work and will be further investigated in the future.

According to the criteria of novel miRNA annotation proposed by Ambros and Berezikov *et al*. [38,50], non-coding small RNAs are defined as genuine miRNAs that fulfill a combination of expression and biogenesis criteria. A novel HAV antigenome-encoded miRNA candidate hav-miR-N1-3p from this study was defined as *bona fide* miRNAs, fulfilling these annotation criteria. In addition, we identified HAV antigenome-encoded hav-miR-N1-3p based on the miRNA biogenesis pathway and biologically silencing activity. Similar to previous results from Hussain *et al*. [24] for WNV encoded miRNA, silencing of Dicer led to a significant decline in hav-miR-N1-3p levels. Further, we noted that knockdown of Drosha reduced hav-miR-N1 expression. These findings further suggested that a cytoplasmic RNA virus could utilize the cellular miRNA processing machinery to produce their own miRNAs. Moreover, the biologically functional activity of the viral miRNA candidate was examined with robust silencing activity. This provides forceful evidence to support the conclusion that the viral miRNA candidate hav-miR-N1-3p was considered genuine miRNA. Overall, the predicted pre-miRNAs can be folded into typical stem-loop structures, and the mature sequence of 22 nt detected in HAV-infected cells. Interestingly, the mature miRNA was derived from the splicing of the predicted pre-miRNA, and knockdown of Dicer and Drosha reduced hav-miR-N1-3p expression. Most importantly, hav-miR-N1-3p silenced luciferase gene expression, both exogenously and endogenously, suggesting the significant functional activity of hav-miR-N1-3p. These results strongly indicated that hav-miR-N1-3p is a genuine miRNA encoded by HAV antigenome. Further investigations will be needed to determine the mechanisms by which HAV antigenome encodes miRNA-like small RNAs as well as the potential biological function of viral miRNA during infection and host-virus interactions. However, the preliminary prediction of hav-miR-N1-3p target genes in host and virus provided some clues for better understanding of the regulatory roles of viral miRNA on host-virus interactions. Indeed, HAV antigenome-encoded hav-miR-N1-3p was completely complementary to the corresponding region of the viral genome, which might lead to targeted cleavage of the viral genome so as to regulate viral replication. HAV displays idiographic interactions with host immune responses [51], which suppress MAVS-mediated signal transduction and block IFN-β induction [52-55]. Thus, genes involved in molecular pathways that are heavily affected by HAV might be regulated by the viral miRNA. Results of cellular target prediction analysis showed that hav-miR-N1-3p had a good sequence complementary with *MAVS* mRNA. This suggests that hav-miR-N1-3p might interact with the *MAVS* gene to regulate cellular antiviral pathways through modulation of *MAVS* gene expression. Without a doubt, more studies are needed to further describe the regulatory roles of viral miRNA.

In summary, we predicted two putative viral pre-miRNAs and six mature miRNAs derived from HAV antigenome. A novel HAV antigenome-encoded miRNA hav-miR-N1-3p was experimentally identified and validated by different and complementary approaches. As a next step, target gene identification for hav-miR-N1-3p will be performed to reveal its potential regulation of viral genes, host genes or both, involved in virus-cell interaction and viral replication in infected cells. Overall, this study is the first to report generation and expression of antigenome derived miRNA in a cytoplasmic picornavirus (hepatitis A virus), and strongly supports the idea that the antigenome of a cytoplasmic RNA virus can naturally encode functional miRNA-like small RNAs through the cellular miRNA processing machinery. Although the exact function of viral miRNA has not yet been elucidated, this study will facilitate further works on its potential biological roles.

## Conclusion

This study demonstrated that the antigenome of a cytoplasmic RNA virus, hepatitis A virus, could be processed into functional miRNAs in infected cells. Our findings provide new evidence for the hypothesis that a cytoplasmic RNA virus can naturally encode miRNAs through cellular miRNA processing machinery.

## Methods

### Cells, viruses, plasmids and reagents

Human lung diploid fibroblastic KMB17 cells (Institute of Medical Biology, CAMS, Kunming, China) were grown in Minimum Essential Medium (MEM) supplemented with 10% bovine serum (Minhai Biotech, Beijing, China) at 37°C in a humid environment containing 5% CO_2_. HEK293T cells were purchased from Thermo Scientific (Cat no. HCL4517) and grown in Dulbecco’s modified Eagle’s medium (DMEM) (high glucose formulation, Gibco, Life Technologies, Grand Island, NY, USA) supplemented with 10% fetal calf serum (Gibco Life Technologies, Grand Island, NY, USA) at 37°C in a humid environment containing 5% CO_2_. Human hepatitis A virus strain H2 (lg10^7.6^ TCID_50_/ml), an attenuated vaccine strain (Institute of Medical Biology, Kunming, CAMS) was prepared in KMB17 cells [56].

The eukaryotic expression plasmid pcDNA6.2-GW/EmGFP-hav-pre-miRNA-50 obtained by inserting the entire viral pre-hav-miR-N1-3p sequence into pcDNA6.2-GW/EmGFP-miR vector (Invitrogen, Carlsbad, CA, USA) was constructed via Gateway cloning using BLOCK-iT™ Pol II miR RNAi Expression Vector Kits (Invitrogen, Carlsbad, CA, USA) according to the manufacturer’s manual. The pmirGLO-hav-miRNA target expression plasmids wild-type (sensor) and mutant type (sensor-mut) were generated by inserting two tandem repeats of the antisense sequence of viral miRNA or cellular miR-154 (or mutated version) into 3' UTR of the luciferase gene of the pmirGLO vector (Promega, Madison, WI, USA), according to the manufacturer’s protocol. All constructs generated were confirmed by sequencing using universal primers (BGI, Guangzhou, China).

Mouse monoclonal anti-Dicer1 antibody 5D12.2 (1:5000 dilution; mouse monoclonal; Millipore Corporation, Billerica, MA, USA), rabbit anti-actin polyclonal antibody (20536-1-Ap; 1:2000 dilution; rabbit polyclonal; Proteintech Group, lnc. Chicago, IL, USA), and appropriate HRP-conjugated anti-mouse and anti-rabbit secondary antibodies (1:10,000 dilution; Proteintech Group, lnc. Chicago, IL, USA) were used for immunoblotting.

### Bioinformatics prediction of the miRNAs

A flowchart describing the computational prediction of putative miRNAs is shown in Figure 1. Briefly, the viral antigenome was scanned for miRNA precursors (pre-miRNA) stem-loop structures using VMir, a computational analyzer program [16,35,36] for prediction of putative pre-miRNAs. Six complete antigenome sequences of different cell-adapted passaged HAV strain H2 (GenBank accession no. EF406358.1, EF406359.1, EF406360.1, EF406361.1, EF406362.1, EF406363.1) were used [56]. VMir predictions were carried out using default parameters. The putative pre-miRNAs that fulfilled filter parameters with VMir score ≥ 150 and window counts ≥ 35 were selected for further assessment. Subsequently, mature miRNA sequences from pre-miNRA stem-loops were predicted (Additional file 1). In order to extend the prediction coverage of the mature miRNAs, we performed two strategies: the MatureBayes tool [37] (http://mirna.imbb.forth.gr/MatureBayes.html) and Bayes-SVM-MiRNA web server v1.0 (http://wotan.wistar.upenn.edu/BayesSVMmiRNAfind/). Default conditions were followed for the MatureBayes tool. Folding energy was set at -15 kcal/mol when using Bayes-SVM-MiRNA web server v1.0; other filter parameters were set to default values.

### Stem-loop RT-PCR analysis and PCR-based directed cloning of the miRNAs

KMB17 cells were infected at a multiplicity of infection (MOI) of 1.0 50% tissue culture infective doses (TCID_50_) /cell of HAV strain H2. Mock-infected cells was used as negative control. At 24 hours post-infection, total RNA was extracted with the Trizol reagent (Invitrogen China Ltd., Shanghai, China) according to manufacturer’s protocol. A highly sensitive and specific stem-loop RT-PCR was used to detect the expression of the candidate miRNAs as described previously [39,57], with minor modifications. Briefly, first strand cDNA of miRNAs were synthesized using stem-loop RT primers (Additional file 3) and Reverse Transcription System (Cat. no. A5001; Promega, Madison, WI, USA), following the manufacturer’s instructions. Then, miRNA cDNAs were amplified by PCR in a mixture including rTaq DNA polymerase (TaKaRa, Dalian, China). The reaction mixture was subjected to 94°C for 5 min, followed by 40 cycles of denaturation at 94°C for 15 sec, annealing at 56°C for 15 sec, and extension at 72°C for 30 sec. A cellular miR-154 was used as a positive control for miRNA size when products were separated by agarose gel electrophoresis. In addition, to determine the specificity of qPCR products, agarose gel electrophoresis analysis and T-A cloning strategy were conducted. The PCR products were analyzed on 4% agarose gel and purified PCR products were subcloned into the pGEM-T vector (Promega, Madison, WI, USA) and sequenced to verify the exact miRNA sequences. KMB17 cells were infected at MOI of 0.5 TCID_50_/cell of HAV. Cells infected at various time points 0, 4, 8, 12, 24, 48, 72, 96, 120 hours were analyzed for time course expression of miRNA. Stem-loop qRT-PCR was performed to quantify miRNA levels using Reverse Trancription System (Cat. no. A5001; Promega, Madison, WI, USA) and GoTaq qPCR kit (Cat. no. A6001; Promega, Madison, WI, USA). Cellular U6 snRNA was as an endogenous control. Relative expression levels were calculated using the 2^-ΔΔCt^ method for infected versus uninfected cells [58].

### RNA interference (RNAi) of the Dicer gene

The siRNA duplexes against the Dicer gene were synthesized according to sequences reported by Moore, *et al.*[59] and Bennasser, *et al.*[60] (Additional file 4). In order to avoid off-target effects, siRNAs were designed for multiple targets of the target gene. All siRNAs used for knockdown of the target gene and scrambled siRNA (a negative control) were chemically synthesized with 2' OME modification by GenePharma (Shanghai, China). siRNAs were dissolved in 0.1% diethylpyrocarbonate (DEPC) treated water to a final concentration of 20 μM and stored at -80°C. KMB17 cells were seeded in 6-well plates one day before transfection. The next day, when the cells reached approximately 50-70% confluence, 100 pmol siRNA against Dicer mRNA or non-specific negative control siRNA were transfected into KMB17 or HEK293T cells using Lipofectamine 2000 (Invitrogen China Ltd., Shanghai, China), according to the manufacturer’s protocol. Seventy-two hours after transfection, siRNAs transfected cells were lysed with RIPA buffer (Pierce, Rockford, IL, USA) containing the protease inhibitor PMSF (Solarbio, Beijing, China). Total cell protein extracts were collected for Western blot analysis.

### Real-time quantitative RT-PCR

The expression levels of viral miRNA and the Dicer gene were analyzed by real-time quantitative RT-PCR (qRT-PCR) with specific primers (Additional files 3 and 5) using the Reverse Trancription System (Cat. no. A5001; Promega, Madison, WI, USA) and GoTaq qPCR kit (Cat. no. A6001; Promega, Madison, WI, USA) according to the manufacturer’s instructions. For viral miRNA, cellular U6 snRNA gene was determined by qRT-PCR in parallel as an internal standard control. Total RNA was analyzed on a CFX96™ Real-Time PCR system (Bio-Rad, CA, USA) using the following program: 94°C for 5 min, followed by 40 cycles of denaturation at 94°C for 15 sec, annealing at 56°C for 15 sec, and extension at 72°C for 30 sec. Relative miRNA abundance was normalized against cellular U6 snRNA content and assessed by the 2^-ΔΔCt^ method [58]. For the Dicer gene, cellular Glyceraldehyde 3-phosphate dehydrogenase (*GAPDH*) mRNA was determined by qRT-PCR in parallel as an endogenous control. Total RNA was analyzed on a CFX96™ Real-Time PCR system (Bio-Rad, CA, USA) under standard conditions using the following program: 94°C for 5 min, followed by 40 cycles of denaturation at 94°C for 15 sec, annealing at 42°C for 15 sec, and extension at 72°C for 30 sec. Relative abundance of target gene mRNA was normalized to cellular *GAPDH* mRNA content and assessed by the 2^-ΔΔCt^ method [58]. Melting curve analysis was also carried out to determine qPCR product specificity. The Ct (cycle threshold) values were determined using default threshold settings. All qRT-PCR assays were performed with three biological replicates and three technical replicates.

### Western blot analysis

Total protein in cell extracts was quantitated by the BCA protein assay Kit (Pierce, Rockford, IL, USA) according to manufacturer’s instructions. Thirty micrograms total protein were resolved on 8% SDS-PAGE gels and transferred onto PVDF membranes (Millipore, Billerica, MA, USA), followed by blocking with 5% non-fat milk at room temperature for 2 hours. Membranes were probed with specific primary antibody overnight at 4°C, followed by incubation with appropriate (anti-mouse or anti-rabbit) HRP-conjugated secondary antibody at room temperature for 1 hour. Protein signals were visualized by ECL chemiluminescence using Immobilon Western HRP Substrate (Millipore Corporation, Billerica, MA, USA) according to the manufacturer’s protocol.

### Luciferase reporter assay

A dual luciferase reporter assay using pmirGLO-hav-miR-N1-3p sensor and pmirGLO-hav-miR-N1-3p-sensor-mut was performed in HEK293T and KMB17 cells. Briefly, HEK293T cells were seeded at approximately 1 × 10^6^ cells per well in a 24-well plate one day before transfection. The next day, 200 ng pmirGLO-hav-miR-N1-3p-sensor or pmirGLO-hav-miR-N1-3p-sensor-mut plasmid were co-transfected with 20 pmol chemically synthesized miRNA mimics (GenePharma, shanghai, China) (Additional file 6) into HEK293T cells with Lipofectamine 2000 (Invitrogen). A cellular miR-154 served as a positive control, and mutated pmirGLO-hav-miR-N1-3p-sensor-mut was used as a negative control. Additionally, KMB17 cells prior to infection with HAV (MOI = 10.0 TCID_50_/cell) were seeded at approximately 1 × 10^6^ cells per well in a 24-well plate one day before transfection. The next day, 200 ng wild type and mutated reporter sensor plasmids were transfected into HAV-infected and mock-infected KMB17 cells. The firefly and *Renilla* luciferase activities were evaluated simultaneously 48 hours post-transfection using the Dual-Glo™ Luciferase Assay System (Promega, Madison, WI, USA) according to the manufacturer’s protocol. Relative luciferase activity was expressed as the ratio of firefly to *Renilla* luciferase activity. The transfections were performed independently, in triplicate.

### Statistical analysis

Values from three independent experiments were analyzed by two-tailed Student’s *t*-test. *P <* 0.05 was considered statistically significant and *P <* 0.01 highly statistically significant.

## Abbreviations

HAV: Hepatitis A virus; miRNA: MicroRNA; pri-miRNA: Primary miRNA; pre-miRNA: Precursor miRNA; mRNA: Messenger RNA; nt: Nucleotide; bp: Base pair; 3' UTR: 3' Untranslated region; WNV: West Nile virus; DENV: Dengue virus; qRT-PCR: Quantitative reverse transcriptase-polymerase chain reaction; PTGS: Post-transcriptional gene silencing.

## Competing interests

The authors declare that they have no competing interests.

## Authors’ contributions

JDS and YZH designed the experiments; JDS, ZQD, BW, MNW, JZ performed the experiments; JDS, JS analyzed data; JDS wrote the manuscript; NZH and HXW provided important reagents and analysis tools; YZH provided overall supervision and financial support and edited the manuscript. All authors read and approved the final manuscript.

## Supplementary Material

Additional file 1: Table S1 Prediction of mature miRNAs derived from HAV antigenome by MatureBayes tool and Bayes-SVM-MiRNA web server v1.0. Click here for file

Additional file 2: Figure S1 Knockdown of Drosha reduced hav-miR-N1-3p expression. Click here for file

Additional file 3: Table S2 Primers for stem-loop (q) RT-PCR of miRNAs. Oligonucleotide sequences for real-time quantitative PCR analysis. Click here for file

Additional file 4: Table S3 Oligonucleotide sequences for RNA interference (RNAi). siRNA duplexes against the Dicer gene. Click here for file

Additional file 5: Table S4 Primers for qRT-PCR of Dicer gene mRNA. Oligonucleotide sequences for real-time quantitative PCR analysis. Click here for file

Additional file 6: Table S5 Synthesized viral miRNA mimics for the luciferase reporter assay. Click here for file
