# Supplementary material for: Identification and validation of a novel microRNA-like molecule derived from a cytoplasmic RNA virus antigenome by bioinformatics and experimental approaches
Source: Virol J. 2014 Jul 1;11:121. doi: 10.1186/1743-422X-11-121 (PMC4087238; doi:10.1186/1743-422X-11-121)
Supplement: Additional file 1: Table S1 — Prediction of mature miRNAs derived from HAV antigenome by MatureBayes tool and Bayes-SVM-MiRNA web server v1.0. [file 1743-422X-11-121-S1.doc]

**Supplemental Table S1. Prediction of mature miRNAs derived from HAV antigenome**

**1. Prediction of MR50 generated-miRNAs**

**1). MatureBayes tool prediction**

**The results of the query**

| **>Precursor hairpin:46:49** | | |
| --- | --- | --- |
| **Sequence** | **AAUCAUGGUUUUAUCAAUGUGAUGAUGAAUGGGACUCUUUCUAAAAAGCGUUUUGG**  **AGACUACAUUCAUUGAACACUGAGUAAAAUCCACUUUCAUGAUU** | |
| **Duplex** | **Position 12 (5' stem)** | **Sequence: AUCAAUGUGAUGAUGAAUGGGA** |
|  | **Position 60 (3' stem)** | **Sequence: UACAUUCAUUGAACACUGAGUA** |
| **Mature 5'stem** | **Position 41 （MR50-2）** | **Sequence: UAAAAAGCGUUUUGGAGACUAC** |
| **Mature 3'stem** | **Position 60 （MR50-1）** | **Sequence: UACAUUCAUUGAACACUGAGUA** |

**2). Bayes-SVM-MiRNA web server v1.0 prediction**

| **Prediction 1** | **1** |
| --- | --- |
| **Name** | **Value** |
| **SVM Score:** | **0.942147** |
| **Sliding window:** | **5-85** |
| **Sequence length:** | **80** |
| **Folding energy:** | **-17.8** |
| **Loop length:** | **6** |
| **Precursor sequence:** | **tggttttatcaatgtgatgatgaatgggactctttctaaaaagcgttttggagactacattcattgaacactgagtaaaa** |
| **miRNA:** | **gatgatgaatgggactctttc (Location: 20-40) （MR50-3）** |
| **Secondary structure: (w/o loop)** | **5' tggtttta-tcaatgtgatgatgaatgggactctttctaaa 3'**  **||||| ||| ||| |||||||| ||||||||**  **3' ---aaaatgagtcacaag-ttacttacatc---agaggttt 5'** |

**2. Prediction of MR35 generated-miRNAs**

**1). MatureBayes tool prediction**

**The results of the query**

| **>Precursor hairpin:51:53** | | |
| --- | --- | --- |
| **Sequence** | **UGGCUCACAUCUUGUUACCAUUGCCUGAUUUAUAGAUCCAAGGUUUUUGAGUUUCUGAUGAACUCUUGCUAUGCAGUCUCUCAAAGGUGACAAAUGAACCA** | |
| **Duplex** | **Position 24 (5' stem)** | **Sequence: CUGAUUUAUAGAUCCAAGGUUU** |
|  | **Position 59 (3' stem)** | **Sequence: GAACUCUUGCUAUGCAGUCUCU** |
| **Mature 5'stem** | **Position 25 (MR35-2)** | **Sequence: UGAUUUAUAGAUCCAAGGUUUU** |
| **Mature 3'stem** | **Position 59 (MR35-1)** | **Sequence: GAACUCUUGCUAUGCAGUCUCU** |

**2). Bayes-SVM-MiRNA web server v1.0 prediction**

| **Prediction 1** | **1** |
| --- | --- |
| **Name** | **Value** |
| **SVM Score:** | **1.51403** |
| **Sliding window:** | **10-90** |
| **Sequence length:** | **80** |
| **Folding energy:** | **-15.5** |
| **Loop length:** | **11** |
| **Precursor sequence:** | **cttgttaccattgcctgatttatagatccaaggtttttgagtttctgatgaactcttgctatgcagtctctcaaaggtga** |
| **miRNA:** | **tgaactcttgctatgcagtct (Location: 58-78) (MR35-3)** |
| **Secondary structure: (w/o loop)** | 5' cttgttaccattgcctgatt-tatagatccaag-gtttt 3'  ||||| ||| |||| ||||| |||| ||||  3' ----agtggaaactctctgacgtatc---gttctcaagt 5' |
