# Supplementary material for: Identification and validation of a novel microRNA-like molecule derived from a cytoplasmic RNA virus antigenome by bioinformatics and experimental approaches
Source: Virol J. 2014 Jul 1;11:121. doi: 10.1186/1743-422X-11-121 (PMC4087238; doi:10.1186/1743-422X-11-121)
Supplement: Additional file 2: Figure S1 — Knockdown of Drosha reduced hav-miR-N1-3p expression. [file 1743-422X-11-121-S2.pdf]

supplemental Fig.S1

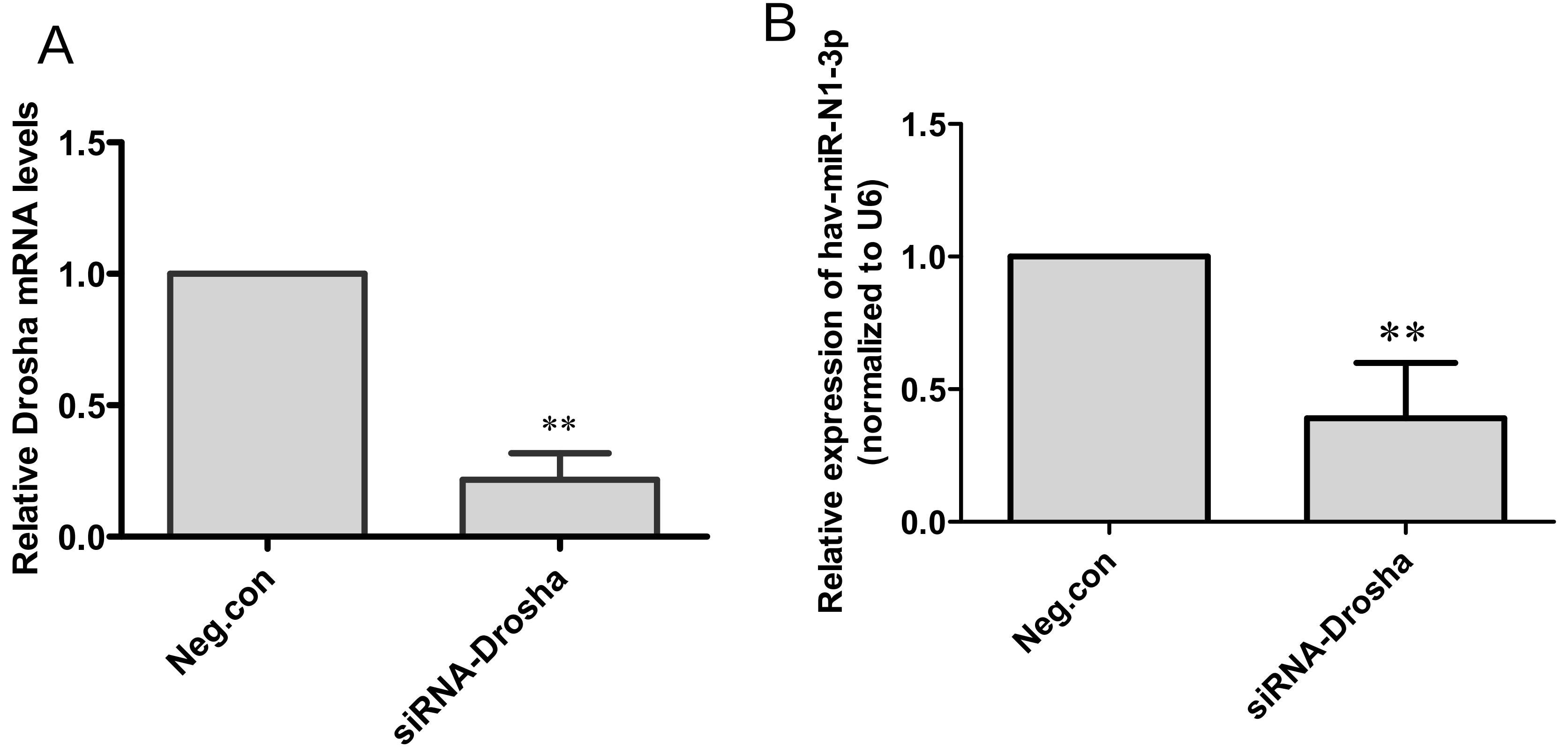

Fig. S1. Knockdown of the Drosha reduced hav-miR-N1-3p expression. (A) qRT-PCR analysis of the Drosha mRNA in Drosha-specific siRNAs treated KMB17 cells and negative control cells. (B) qRT-PCR analysis of hav-miR-N1-3p in KMB17 cells infected with HAV after the Drosha knockdown. Data were normalized against U6 snRNA. Values are described as means from triplicate experiments. The significant differences of data are indicated with ** at $P<0.01$.

Primers and siRNAs against Drosha mRNA

primer	primer sequence (5'- 3')	product Length (bp)
Drosha-Fwd	CCCTCCGGGCTATTCTCAC	189
Drosha-Rev	TGGTCATCATAGTGTTTCAGCCT	
GAPDH-Fwd	GGAGCGAGATCCCTCCAAAAT	197
GAPDH-Rev	GGCTGTTGTCATACTTCTCATGG	

QPCR conditions:

94℃, 5min → 40× （94 ℃, 15s → 42 ℃, 15s, → 72 ℃, 30s ）→ melt curve analysis

siRNA	sequence (5'- 3')
Drosha - siRNA-1	UAA AGU AGC UGG AAU GAU G
Drosha - siRNA-2	GAA UAU GGU UGU UUG AAG A
Drosha - siRNA-3	ACA CAG CAG UUG UCU UAA A
Drosha - siRNA-4	GAA UAU CGA UCC UAU GUU C
Non-silencing control	UUC UCC GAA CGU GUC ACG U

siRNA duplexes with the sense sequences indicated were purchased from Genpharma (Shanghai, China). In all cases, siRNA duplexes had dT overhangs.
