# Supplementary material for: Identification and validation of a novel microRNA-like molecule derived from a cytoplasmic RNA virus antigenome by bioinformatics and experimental approaches
Source: Virol J. 2014 Jul 1;11:121. doi: 10.1186/1743-422X-11-121 (PMC4087238; doi:10.1186/1743-422X-11-121)
Supplement: Additional file 3: Table S2 — Primers for stem-loop (q) RT-PCR of miRNAs. Oligonucleotide sequences for real-time quantitative PCR analysis. [file 1743-422X-11-121-S3.doc]

**Supplemental Table S2. Primers used for miRNA stem-loop (q)RT-PCR**

| **miRNA** | **primer** | **primer sequence (5**'**-3**'**)** |
| --- | --- | --- |
| MR50-1 | RT-primer | gtcgtatccagtgcagggtccgaggtattcgcactggatacgac-TACTCAGT |
|  | Fwd-primer | TGCTGCTACATTCATTGAACAC |
|  | Rev-primer | gtgcagggtccgaggt |
| MR50-2 | RT-primer | gtcgtatccagtgcagggtccgaggtattcgcactggatacgac-GTAGTCTC |
|  | Fwd-primer | TATTAcTAAAAAGCGTTTTG |
|  | Rev-primer | gtgcagggtccgaggt |
| MR50-3 | RT-primer | gtcgtatccagtgcagggtccgaggtattcgcactggatacgac-GAAAGAGT |
|  | Fwd-primer | GCCGATGATGATGAATGGG |
|  | Rev-primer | gtgcagggtccgaggt |
| MR35-1 | RT-primer | gtcgtatccagtgcagggtccgaggtattcgcactggatacgac-AGAGACTG |
|  | Fwd-primer | CACAGAGAACTCTTGCTATG |
|  | Rev-primer | gtgcagggtccgaggt |
| MR35-2 | RT-primer | gtcgtatccagtgcagggtccgaggtattcgcactggatacgac-AAAACCTT |
|  | Fwd-primer | TGGTGTTGATTTATAGATCC |
|  | Rev-primer | gtgcagggtccgaggt |
| MR35-3 | RT-primer | gtcgtatccagtgcagggtccgaggtattcgcactggatacgac-CACCTTTG |
|  | Fwd-primer | GCGCGCCTATGCAGTCTCT |
|  | Rev-primer | gtgcagggtccgaggt |
| miR-154 | RT-primer | gtcgtatccagtgcagggtccgaggtattcgcactggatacgac-CGAAGGCA |
|  | Fwd-primer | TGGTTGTAGGTTATCCGTGTTG |
|  | Rev-primer | gtgcagggtccgaggt |
| U6 | RT-primer | AACGCTTCACGAATTTGCGT |
|  | Fwd-primer | CTCGCTTCGGC AGCACA |
|  | Rev-primer | AAACGCTTCACGAATTTGCGT |
