# Supplementary material for: Identification and validation of a novel microRNA-like molecule derived from a cytoplasmic RNA virus antigenome by bioinformatics and experimental approaches
Source: Virol J. 2014 Jul 1;11:121. doi: 10.1186/1743-422X-11-121 (PMC4087238; doi:10.1186/1743-422X-11-121)
Supplement: Additional file 4: Table S3 — Oligonucleotide sequences for RNA interference (RNAi). siRNA duplexes against the Dicer gene. [file 1743-422X-11-121-S4.doc]

**Supplemental Table S3. siRNA oligonucleotides against Dicer gene**

| **Gene Targeted** | **siRNA** | **siRNA sequence (5**'**-3**'**)*** |
| --- | --- | --- |
| Dicer | Dicer- siRNA-1 | UAA AGU AGC UGG AAU GAU G |
|  | Dicer- siRNA-2 | GAA UAU GGU UGU UUG AAG A |
|  | Dicer- siRNA-3 | ACA CAG CAG UUG UCU UAA A |
|  | Dicer- siRNA-4 | GAA UAU CGA UCC UAU GUUC |
|  | Dicer- siRNA-5 | UGCUUGAAGCAGCUCUGGA |
| Non-silencing control | | CAG UCG CGU UUG CGA CUG G |

*RNA duplexes with the sense sequences indicated were purchased from Genpharma. For Dicer, multiple siRNAs were used simultaneously to increase the knockdown efficiency. In all cases RNA duplexes had dT overhangs.
