# Supplementary material for: Identification and validation of a novel microRNA-like molecule derived from a cytoplasmic RNA virus antigenome by bioinformatics and experimental approaches
Source: Virol J. 2014 Jul 1;11:121. doi: 10.1186/1743-422X-11-121 (PMC4087238; doi:10.1186/1743-422X-11-121)
Supplement: Additional file 5: Table S4 — Primers for qRT-PCR of Dicer gene mRNA. Oligonucleotide sequences for real-time quantitative PCR analysis. [file 1743-422X-11-121-S5.doc]

**Supplemental Table S4. Primers used for qRT-PCR of Dicer gene mRNA**

| **Primer** | **Primer Sequence (5**'**-3**'**)** | **Product Length (bp)** |
| --- | --- | --- |
| Dicer-Forward | TGCTATGTCGCCTTGAATGTT | 114 |
| Dicer-Reverse | AATTTCTCGATAGGGGTGGTCTA |  |
| GAPDH-Forward | TCGACAGTCAGCCGCATCT | 87 |
| GAPDH-Reverse | CCGTTGACTCCGACCTTCA |  |
