# Supplementary material for: Identification and validation of a novel microRNA-like molecule derived from a cytoplasmic RNA virus antigenome by bioinformatics and experimental approaches
Source: Virol J. 2014 Jul 1;11:121. doi: 10.1186/1743-422X-11-121 (PMC4087238; doi:10.1186/1743-422X-11-121)
Supplement: Additional file 6: Table S5 — Synthesized viral miRNA mimics for the luciferase reporter assay. [file 1743-422X-11-121-S6.doc]

**Supplemental Table S5. Synthesized viral miRNA mimic duplexes for luciferase reporter assay.**

| **HAV miRNA** | **miRNA mimic sequence (5'-3')** |
| --- | --- |
| hav-miR-N1-3p | UACAUUCAUUGAACACUGAGUA |
|  | UCAGUGUUCAAUGAAUGUAUUG |
| Cellular miR-154 | UAGGUUAUCCGUGUUGCCUUCG AAGGCAACACGGAUAACCUAUU |
| Negativ control | UUCUCCGAACGUGUCACGUTT  ACGUGACACGUUCGGAGAATT |
